# Immune Checkpoint Receptor Expression Profiles of MAIT Cells in Moderate and Severe COVID‐19

**DOI:** 10.1111/sji.70008

**Published:** 2025-02-20

**Authors:** Matyas Meggyes, David U. Nagy, Ildiko Toth, Timoteus Feik, Beata Polgar, Iyad Saad Al Deen, David Sipos, Laszlo Szereday, Agnes Peterfalvi

**Affiliations:** ^1^ Department of Medical Microbiology and Immunology Medical School, University of Pecs Pecs Hungary; ^2^ Janos Szentagothai Research Centre Pecs Hungary; ^3^ Institute of Geobotany/Plant Ecology, Martin‐Luther‐University Halle (Saale) Germany; ^4^ Department of Anesthesiology and Intensive Therapy Medical School, University of Pecs Pecs Hungary; ^5^ 1st Department of Medicine, Division of Infectious Diseases Medical School, University of Pecs Pecs Hungary; ^6^ Department of Laboratory Medicine Medical School, University of Pecs Pecs Hungary

**Keywords:** COVID‐19, cytokine storm, disease severity, flow cytometry, immune checkpoints, MAIT cells, SARS‐CoV‐2, viral infection

## Abstract

MAIT cells are one of the largest unconventional T cell populations and, recruited to the site of infection, play both protective and pathogenic roles during pulmonary viral infections. MAIT cell activation patterns change significantly during COVID‐19, with a notable decrease in their frequency in peripheral blood of severe cases. In the present study, we aimed to investigate the expression profiles of various immune checkpoint pathways on MAIT, MAIT‐like and non‐MAIT cells in moderate and severe COVID‐19 patients undergoing cytokine storm. Despite numerous studies comparing MAIT cell characteristics based on COVID‐19 disease severity, none have delved into the critical differences in MAIT cell immune checkpoint profiles between moderate and severe COVID‐19 patients, all experiencing a cytokine storm. Flow cytometry was used to analyse peripheral blood mononuclear cells from a cohort of 35 patients, comprising 18 moderate and 17 severe cases, alongside 14 healthy controls. Our investigation specifically focuses on severe COVID‐19 presentations, revealing a marked deletion of MAIT cells. Further exploration into the regulatory dynamics of MAIT cell functionality reveals shifts in the expression profiles of critical immune checkpoint receptors, notably PD‐1 and CD226. In severe COVID‐19 patients, MAIT cells showed a significant decrease in the expression of CD226, whereas MAIT‐like and non‐MAIT cells demonstrated a significant increase in the expression of PD‐1 compared to healthy individuals. The expression of the TIGIT receptor remained unaltered across all investigated groups. Our findings contribute to the existing knowledge by elucidating the changes in MAIT cell subpopulations and their potential role in COVID‐19 disease severity.

## Graphical abstract

Severe COVID‐19 leads to a marked depletion of MAIT cells and dysregulation of immune checkpoint receptor expression. Our study reveals decreased CD226 expression in MAIT cells and increased PD‐1 expression in MAIT‐like and non‐MAIT cells, highlighting their role in disease severity and immune dysfunction during cytokine storms.

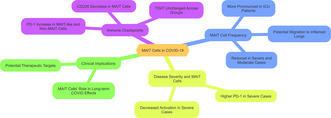

## 1 Introduction

The ongoing COVID‐19 pandemic is causing an unprecedented global health crisis, in which not only demographic and socioeconomic factors, underlying health conditions such as obesity and diabetes, or gender affects disease severity and mortality [1] but also genetic predisposition contributes to the development of severe COVID‐19 [2]. Genome‐wide association studies identified several genes encoding pro‐inflammatory chemokine receptors and molecules from the type I interferon pathway associated with developing severe COVID‐19 [2]. Therefore, the distribution of immune cells within tissues and the innate immune system's responsiveness to infection could be crucial determinants in mitigating severe outcomes of COVID‐19.

One severe complication of COVID‐19 is the cytokine storm, which occurs due to an excessive increase in inflammatory mediators, especially pro‐inflammatory interleukins such as IL‐1β, IL‐6 and TNF‐α [3, 4]. Although cytokines are essential for combating infections, an overactive immune response can lead to tissue damage, multiorgan failure and even death [3]. However, a cytokine storm does not uniformly correlate with disease severity in all patients.

In humans, mucosal‐associated invariant T (MAIT) cells are one of the largest unconventional αβ T cell population, normally found at levels of 1%–10% of T cells in the peripheral blood and the lung [5, 6]. Primarily resident in mucosal tissues, including the lungs, they can rapidly respond upon activation by producing inflammatory cytokines, such as IFN‐γ, TNF‐α, IL‐22 and IL‐17A, and exerting cytotoxic activity [5]. Recent studies indicate that MAIT cells can play both protective and pathogenic roles during pulmonary viral infections [5, 7, 8]. They also contribute to the inflammatory response and antimicrobial immunity [9]. Moreover, they can fine‐tune the host immune response's intensity and quality, shaping the magnitude of the adaptive immune response. However, MAIT cells also participate in the resolution of inflammation, including tissue repair and regeneration, which are crucial steps during acute respiratory distress syndrome [10, 11]. Due to their versatile functions and presence in mucosal tissues, they may serve as important factors in the immunopathology induced by SARS‐CoV‐2. Recently, research groups observed certain phenotypic and functional changes in MAIT cells in patients with COVID‐19 that were associated with the severity of the disease [9, 12, 13]. These studies have revealed that MAIT cell numbers in the peripheral blood decreased with disease severity. The increase in cell cytotoxicity, chemotaxis and apoptosis levels of these cells was consistent with disease severity and displayed the highest levels in patients with severe disease.

Immune checkpoint molecules are expressed across immune cell subsets, including MAIT cells [14, 15]. Coinhibitory immune checkpoint molecules, including CTLA‐4, TIM‐3, TIGIT, PD‐1 and LAG‐3, usually inhibit immune responses by negatively regulating immune cell signalling pathways to prevent immune injury [16, 17]. In contrast, co‐stimulatory immune checkpoints, like CD226, enhance immune responses by promoting cellular cytotoxicity and cytokine production, thereby modulating immune surveillance and effector functions [18, 19].

The above findings showed that besides MAIT cells' involvement in the immune response against SARS‐CoV‐2, they also possibly engage in COVID‐19 immune damage. As such, investigating the role and regulation of MAIT cell subpopulations in COVID‐19 may offer insights into disease severity, immune responses and potential therapeutic targets for managing the infection. To address this issue, our present study focuses on various immune checkpoint pathways expressed by MAIT, MAIT‐like and non‐MAIT cells in moderate and severe COVID‐19 patients undergoing a cytokine storm.

## 2 Materials and Methods

### 2.1 Ethics Statement

Written informed consent was obtained from all subjects involved in the study. To ensure compliance with EU‐GDPR and relevant data protection regulations, the blood collection process followed strict scientific protocols. This guaranteed that all personal and health data were treated anonymously, securely and confidentially. No individual information was provided to the research team for subsequent demographic analysis.

The study was conducted in accordance with the Declaration of Helsinki and approved by the Ethics Committee of the University of Pécs, Hungary (ethical registration number: 8759‐PTE/2021).

### 2.2 Study Cohort and Patient Recruitment

The current study utilised a cohort comprising 35 patients (Table 1) admitted to the University of Pécs, Pécs, Hungary, during the third pandemic wave dominated by the Delta variant, from 23 April 2021 to 7 December 2021. The cohort comprised 18 patients admitted to the infectious disease unit (IDU) with moderate disease and 17 patients requiring intensive care unit (ICU) admission due to severe COVID‐19 manifestations, with a noteworthy ICU mortality rate of 64%. The ICU patients were notably older than those in the other groups (age, mean and years for ICU: 66 ± 14, IDU: 52.2 ± 11 and healthy controls: 44 ± 13). Gender distribution did not significantly differ among the groups. Obesity, as indicated by BMI, showed no significant differences between IDU and ICU patients. Hypertension prevalence was significantly higher in ICU patients (82.4%) compared to IDU patients (33.3%) (*p* < 0.01). Other comorbidities such as heart disease, chronic lung disease, chronic kidney disease and diabetes were more prevalent in ICU patients but did not show statistically significant differences between the groups. The time elapsed from symptom onset to admission was similar between IDU and ICU patients, with no significant differences observed. Of the 35 patients in the study, 24 recovered and were discharged, whereas 11, all from the severe group, died during hospitalisation. None of the IDU patients were deceased. Moreover, healthy controls (non‐vaccinated and without anti‐SARS‐CoV‐2 antibodies) were also recruited.

**TABLE 1:** Demographic characteristics and comorbidities of patients on admission.

Variables	Healthy controls (*n* = 14)	IDU patients (*n* = 18)	ICU patients (*n* = 17)	*p*
Age, mean, years (±SD)	44 ± 13	52.2 ± 11	**66 ± 14**	**< 0.05 versus HC** **< 0.05 versus IDU**
Females/males	6/8	6/12	6/11	NS
Obesity (BMI, median) (min., max.)	NA	27.78 (19.11, 45.2)	29.4 (21.48, 71.78)	NS
Comorbidities				
Hypertension (*n*, %)	0	6 (33.3)	**14 (82.4)**	**< 0.01 versus IDU**
Heart disease (*n*, %)	0	0	3 (17.6%)	NS
Chronic lung disease (*n*, %)	0	1 (5.5)	0	NS
Chronic kidney disease (*n*, %)	0	0	2 (11.7)	NS
Diabetes (*n*, %)	0	2 (11.1)	6 (35.3)	NS
Days elapsed between the onset of symptoms and admission	—	6.7 ± 3.6	5.4 ± 4.1	NS

*Note:* Data are presented as mean ± SD, median (minimum, maximum) or numbers and percentages. Differences were considered significant when the *p* value was less than or equal to 0.05. Comparison of IDU versus ICU patients on the day of admission versus healthy controls. Significant results are presented in bold.

Abbreviations: BMI, body mass index; HC, healthy control; ICU, intensive care unit; IDU, infectious disease unit; NA, data not available; NS, not significant.

### 2.3 Diagnostic Methods

SARS‐CoV‐2 infection was confirmed upon admission through RT‐PCR tests employing LightMix Modular E‐ and N‐gene kits (Roche Diagnostics GmbH, Mannheim, Germany). Patient inclusion criteria were based on the quick COVID‐19 cytokine storm score delineated by Cappanera et al., incorporating parameters such as lymphocyte count, d‐dimer levels, LDH activity, ferritin and CRP [20]. Peripheral venous blood collection was performed on the participant's first day of admission to the IDU or ICU.

### 2.4 Clinical Assessment

Disease severity and clinical prognosis were evaluated utilising the Sequential Organ Failure Assessment (SOFA) score, Simplified Acute Physiology Score (SAPS) and computed tomography (CT) scores assessing lung involvement. Additionally, comparative analyses were performed using blood samples from 14 healthy, uninfected individuals as controls, who were screened negative for anti‐SARS‐CoV‐2 antibodies (anti‐spike and anti‐nucleocapsid) tested with a Roche Cobas automated clinical immunochemistry analyser.

### 2.5 PBMC Separation and Cryopreservation

Peripheral blood mononuclear cells (PBMCs) were separated from heparinised venous blood on the density gradient of Ficoll‐Paque (GE‐Healthcare, USA). The PBMC fraction was collected and washed in complete Rosewell Park Memorial Institute 1640 (RPMI, Lonza, Switzerland) medium supplemented with 10% foetal calf serum (FCS, Lonza, Switzerland). Following the determination of the cell number, the samples were centrifuged and resuspended in inactivated human AB serum containing 10% DMSO (Sigma‐Aldrich, USA) for cryoprotection. The samples were stored at −80°C in a mechanical freezer for further flow cytometric investigation.

### 2.6 Thawing and Flow Cytometric Analyses

On the day of examinations, the cryopreserved samples were thawed in a 37°C water bath and resuspended in the RPMI 1640 medium. They were washed twice in phosphate‐buffered saline (PBS) to eliminate residual DMSO content.

**FIGURE 1:**
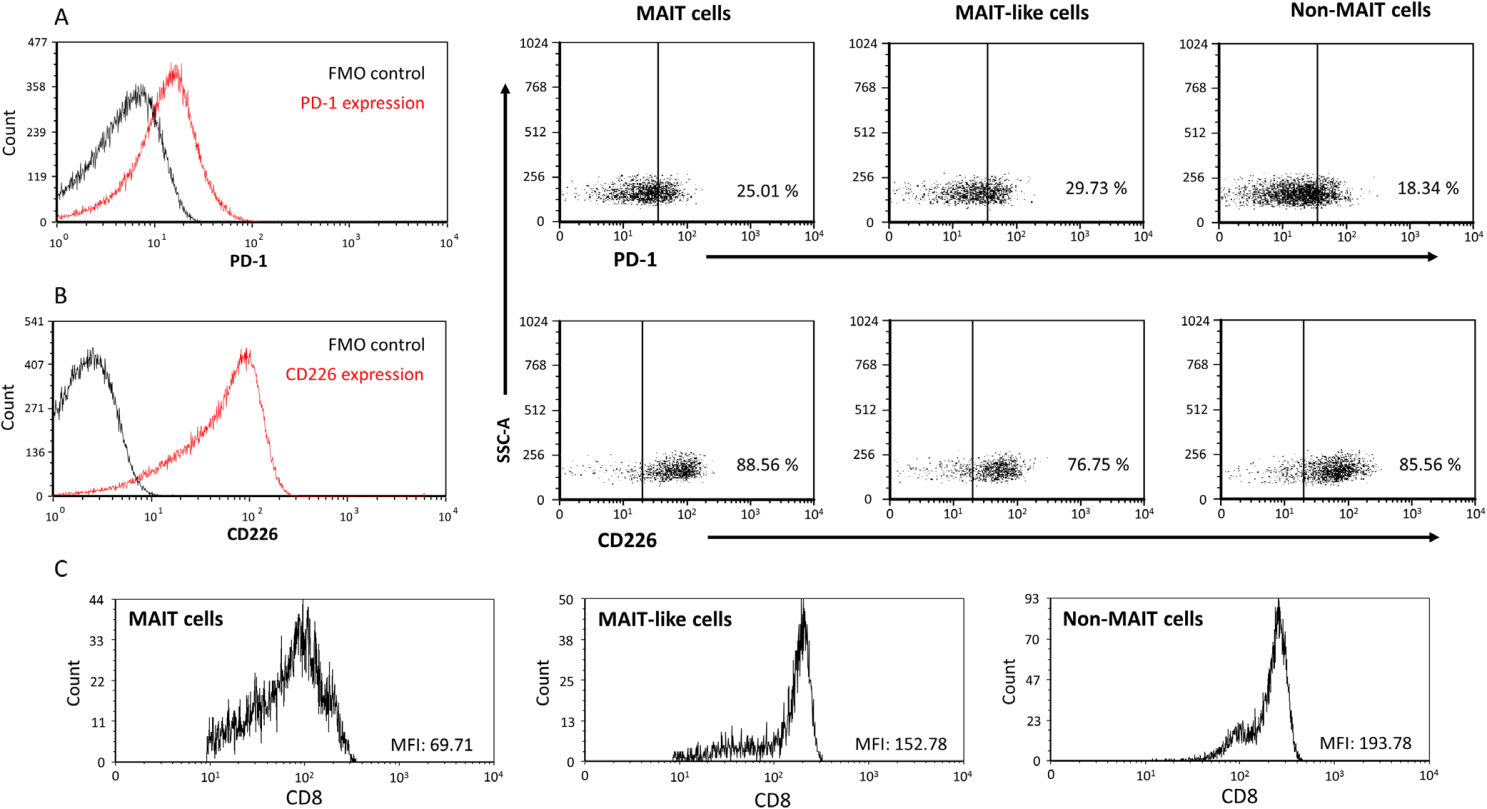
Analysis of the surface receptor positivity by flow cytometry. Representative FACS plots and histograms show the expression of PD‐1 (A) and CD226 (B) receptors by MAIT‐, MAIT‐like and non‐MAIT cells. FMO control was used to determine the positivity of the examined receptors. Representative histograms show the mean fluorescent intensity of the CD8 receptor by the investigated MAIT cell subpopulations (C).

Before surface labelling of thawed PBMCs, Fc receptor‐expressing monocytes were blocked with Human TruStain FcX Blocking Solution (Biolegend, USA) for 10 min. For flow cytometric staining, a combination of fluorochrome‐conjugated monoclonal antibodies (Table 2) was added to 106 PBMCs for 30 min at room temperature in complete darkness. Next, the cells were washed in PBS and resuspended in 300 μL PBS (BioSera, France) containing 1% paraformaldehyde (PFA) and stored at 4°C in complete darkness until analysis using FACS. The relative expression levels of the investigated IC molecules on various cell surfaces were quantified using mean fluorescent intensity (MFI) values. Flow cytometric measurements were performed using a BD FACS Canto II flow cytometer (BD Immunocytometry Systems, Belgium) and the BD FACS Diva V6 software (BD Biosciences, USA) for data acquisition. Flow cytometric data were analysed using FCS Express V4 software (De Novo Software, USA). To determine the positivity of the examined receptors, fluorescent minus one (FMO) control was applied (Figure 1).

**TABLE 2:** Fluorochrome‐conjugated monoclonal antibodies were used in the study.

Antigen	Format	Clone	Isotype	Company	CAT
CD3	BV510	UCHT1	Mouse BALB/c IgG1, κ	BD Biosciences	563109
CD8	APC‐H7	SK1	Mouse BALB/c IgG1, κ	BD Biosciences	560179
CD161	APC	191B8	Mouse IgG2a κ	Miltenyi Biotec.	130113590
CD226	BV421	DX11	Mouse BALB/c IgG1, κ	BD Biosciences	742493
PD‐1	PE	PD1.3	Mouse IgG2b	Beckman Coult.	B30634
TIGIT	PE	A1553G	Mouse IgG2a, κ	Biolegend	372704
Va7.2	FITC	3C10	Mouse IgG1, κ	Biolegend	351704

### 2.7 Statistical Analyses

One‐way ANOVA was used to test the main effect of the group on the measured response variables. Before the analysis, response variables were loge transformed. Decisions on the transformation of variables depended on visual inspection of “model‐checking plots” in R for the models with transformed versus untransformed variables. These plots allow checking assumptions about the normality of residuals and variance homogeneity. For pairwise comparisons of ANOVA tests, Tukey post hoc tests were performed to compare combinations to each other [21].

## 3 Results

### 3.1 Altered Frequency of MAIT Cells in the Blood of Patients With Severe and Moderate COVID‐19 Disease

We initiated our investigation into the immune cell response in SARS‐CoV‐2 infection by examining the frequency of MAIT cells in peripheral blood samples obtained from patients with severe (ICU patients), patients with moderate (IDU patients) COVID‐19 and uninfected healthy controls.

**FIGURE 2:**
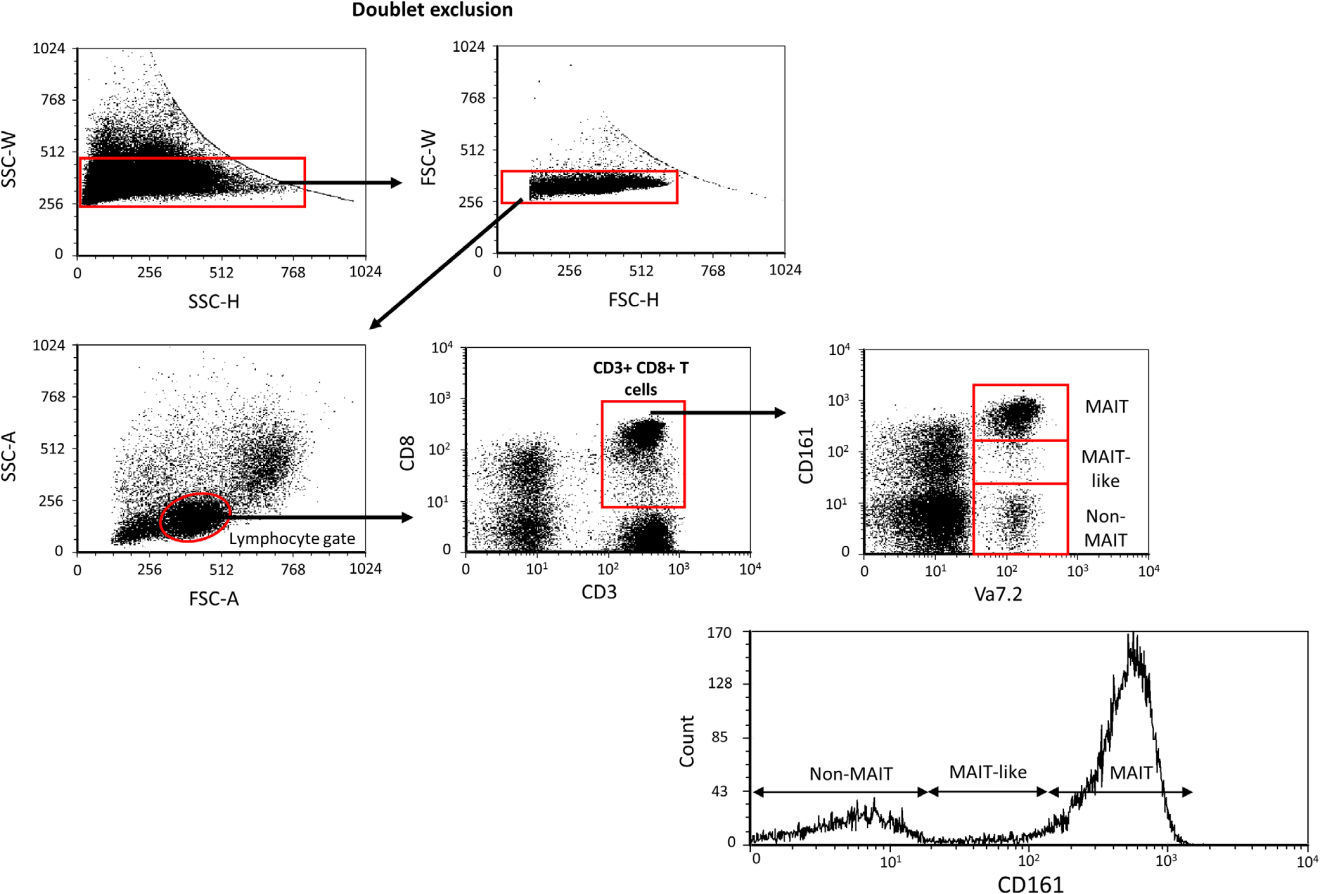
Gating strategy to identify MAIT, MAIT‐like and non‐MAIT cell populations. Following a doublet exclusion, the lymphocyte gate was determined using forward (FSC‐A) and side scatter (SSC‐A) parameters. From the lymphocyte gate, CD3+/CD8+ T cells were selected, and using CD161 and TCRVα7.2 markers, MAIT cells (Vα7.2/CD161++), MAIT‐like cells (Vα7.2/CD161+) and non‐MAIT cells (Vα7.2/CD161‐) were categorised [22, 23, 24, 25].

MAIT cell populations, including MAIT, MAIT‐like and non‐MAIT cells, were characterised in peripheral blood using flow cytometric analysis with fluorochrome‐conjugated monoclonal antibodies targeting CD3, CD8, Va7.2 and CD161. The gating strategies employed for identification are presented in Figure 2.

**FIGURE 3:**
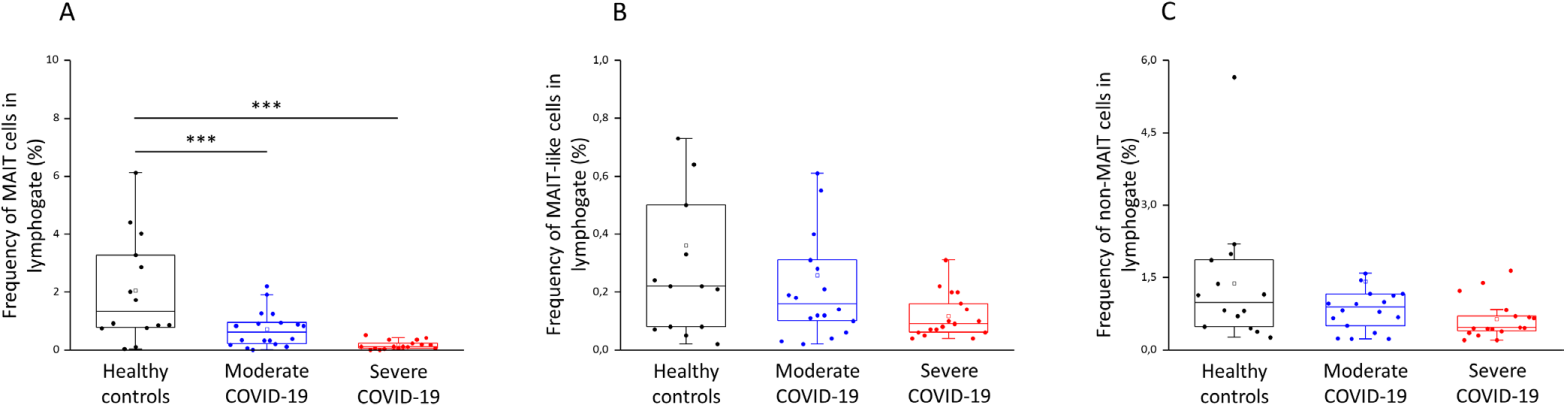
Frequencies of MAIT, MAIT‐like and non‐MAIT cell populations in patients with moderate or severe COVID‐19 and healthy controls. The determined frequencies of the circulating MAIT (A), MAIT‐like (B) and non‐MAIT (C) cell subsets in the peripheral blood of patients with moderate or severe COVID‐19 and healthy controls. The solid bars represent medians of 14, 18 and 17 determinations, respectively. The boxes indicate the interquartile ranges; the whiskers represent the variability of the minimum, maximum and any outlier data points in comparison to the interquartile range. The middle square within the box represents the mean value. Significant differences with *p*‐values < 0.01*** are indicated.

The frequency of the CD3+/CD8+/Va7.2+/CD161++ MAIT population was significantly reduced in the peripheral blood of IDU and ICU patients compared to healthy individuals (Figure 3A). Notably, the frequencies of MAIT‐like (CD3+/CD8+/Va7.2+/CD161+) and non‐MAIT cells (CD3+/CD8+/Va7.2+/CD161‐) were not significantly affected by SARS‐CoV‐2 infection (Figure 3B,C) [22, 23, 24, 25]. Numerous studies have demonstrated that percentage data are crucial for analysing and understanding immune cell population dynamics, providing valuable insights even when absolute cell counts are not available [26, 27, 28].

Although our study focuses on proportional data for MAIT, MAIT‐like and non‐MAIT Va7.2+ cells, we acknowledge that changes in these proportions may partly reflect alterations in the total number of MAIT cells. Specifically, a loss of Va7.2+ MAIT cells could increase the relative proportion of MAIT‐like and non‐MAIT cells, as they share the same TCR Va7.2 marker. Consequently, caution is warranted when interpreting proportional shifts as definitive evidence of functional or population dynamics.

**FIGURE 4:**
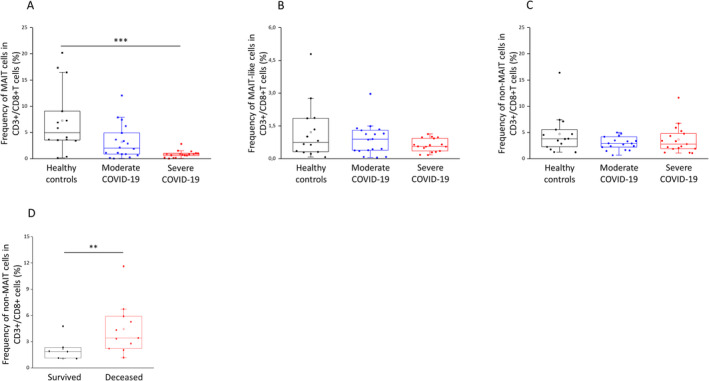
Frequencies of MAIT, MAIT‐like and non‐MAIT cell populations within the CD3+/CD8+ subpopulations in patients with moderate or severe COVID‐19 and healthy controls. The determined frequencies of the circulating MAIT (A), MAIT‐like (B), and non‐MAIT (C) cell subsets within the CD3+/CD8+ subset in the peripheral blood of patients with moderate or severe COVID‐19 and healthy controls. The frequency of the circulating non‐MAIT cell subset within the CD3+/CD8+ subset in the peripheral blood of survived and deceased patients (D). The solid bars represent medians of 14, 18 and 17 determinations, respectively. The boxes indicate the interquartile ranges, the whiskers represent the variability of the minimum, maximum and any outlier data points in comparison to the interquartile range. The middle square within the box represents the mean value. Significant differences with *p*‐values <0.01***, < 0.03** are indicated.

While investigating the frequency of these subpopulations in the CD8+ T cell gate, we revealed a significant decrease in the frequency of MAIT cells in patients with severe disease compared to healthy individuals (Figure 4A). Although MAIT‐like and non‐MAIT cell frequencies were not altered in the CD8+ T cell gate (Figure 4B,C), we found a significant increase in non‐MAIT cells in deceased ICU patients compared to patients with severe disease who survived COVID‐19 infection (Figure 4D).

**FIGURE 5:**
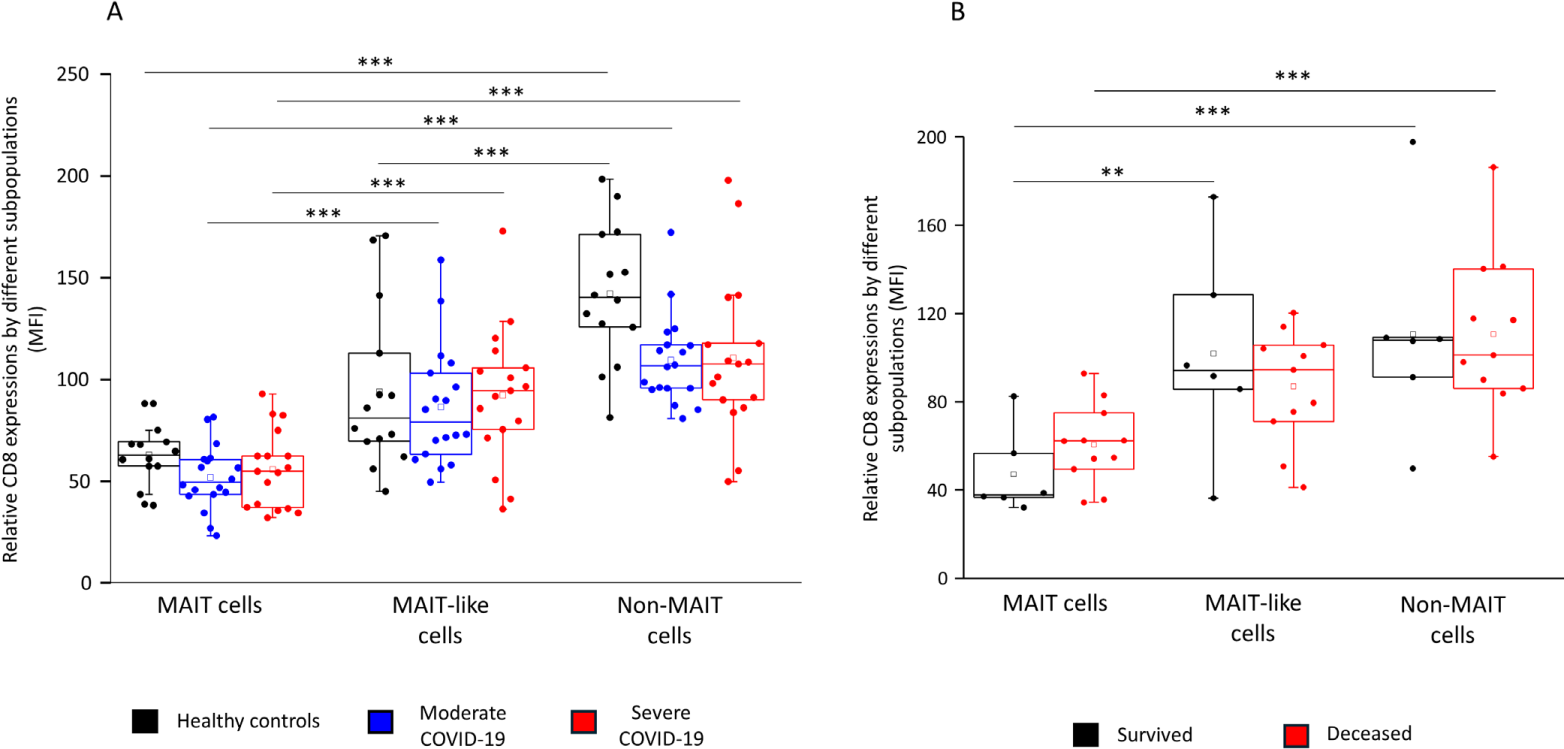
Relative CD8 receptor expression by MAIT, MAIT‐like and non‐MAIT cell populations. The relative expression of the CD8 surface molecule of MAIT, MAIT‐like and non‐MAIT cells in patients with moderate or severe COVID‐19 and healthy controls (A). The relative expression of the CD8 surface molecule of MAIT, MAIT‐like and non‐MAIT cells in survived and deceased patients (B). The solid bars represent medians of 14, 18 and 17 determinations, respectively. The boxes indicate the interquartile ranges; the whiskers represent the variability of the minimum, maximum and any outlier data points in comparison to the interquartile range. The middle square within the box represents the mean value. Significant differences with *p* < 0.01*** and < 0.03** are indicated.

We further investigated the relative surface CD8 expression by the three cell subpopulations, and a significant decrease was observed in MAIT cells compared to MAIT‐like and non‐MAIT cells. In contrast, neither of the cell populations showed any difference between healthy controls and patients with moderate or severe COVID‐19 disease (Figure 5A). None of the investigated cell populations showed any difference regarding relative CD8 expression among deceased ICU patients compared to patients with severe disease who survived COVID‐19 infection (Figure 5B).

### 3.2 Immune Checkpoint Expression by MAIT, MAIT‐Like and Non‐MAIT Cells in the Blood of Patients With Severe and Moderate COVID‐19 Disease

PD‐1 (inhibitory), CD226 (activating) and TIGIT (inhibitory) receptor expressions were measured on the surface of the investigated subpopulations using flow cytometry.

**FIGURE 6:**
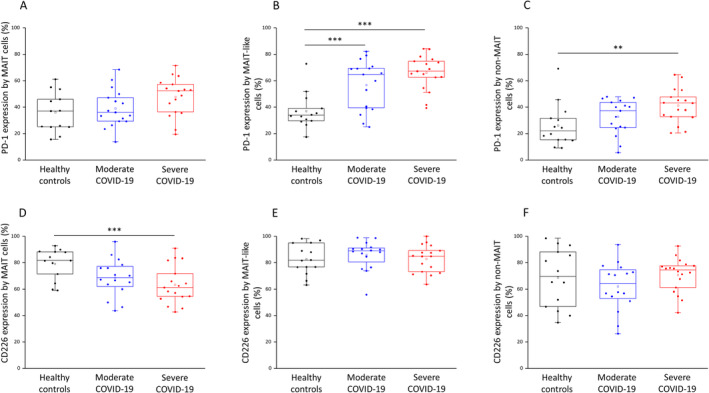
Immune checkpoint expressions by MAIT, MAIT‐like and non‐MAIT cell populations in patients with moderate or severe COVID‐19 and healthy controls. The surface expression of the PD‐1 receptor by MAIT (A), MAIT‐like (B) and non‐MAIT (C) cell populations in patients with moderate or severe COVID‐19 and healthy controls. The surface expression of the CD226 receptor by MAIT (D), MAIT‐like (E) and non‐MAIT (F) cell populations in patients with moderate or severe COVID‐19 and healthy controls. The solid bars represent medians of 14, 18 and 17 determinations, respectively. The boxes indicate the interquartile ranges; the whiskers represent the variability of the minimum, maximum and any outlier data points in comparison to the interquartile range. The middle square within the box represents the mean value. Significant differences with *p* < 0.01*** and < 0.03** are indicated.

In ICU patients, MAIT‐like and non‐MAIT cells showed a significantly increased expression of PD‐1 (Figure 6B,C), whereas the expression levels of the CD226 receptor remained unchanged compared to healthy controls (Figure 6E,F). Similar to ICU patients, PD‐1 expression by MAIT‐like cells significantly increased in patients with moderate disease compared to healthy individuals (Figure 6B). On the other hand, MAIT cells from ICU patients exhibited a significant decrease in the expression of the activating CD226 receptor compared to healthy controls (Figure 6D). No significant difference was observed regarding the expression of the inhibitory TIGIT receptor in any investigated group (data not shown).

**FIGURE 7:**
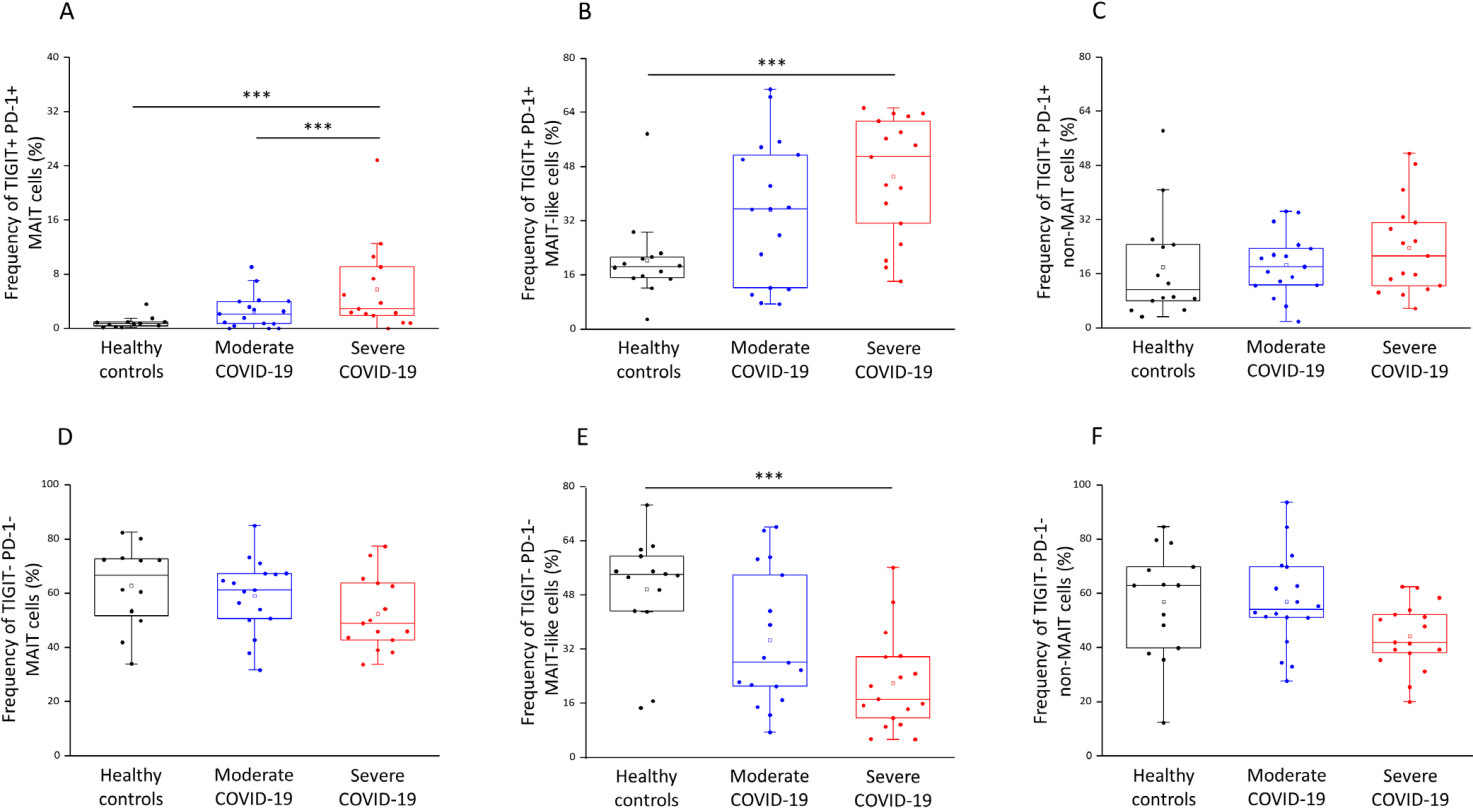
Frequencies of TIGIT/PD‐1 double‐positive and negative MAIT, MAIT‐like and non‐MAIT cell populations in patients with moderate or severe COVID‐19 and healthy controls. The calculated frequencies of the TIGIT and PD‐1 double‐positive MAIT (A), MAIT‐like (B) and non‐MAIT (C) cell subsets in the peripheral blood of patients with moderate or severe COVID‐19 and healthy controls. Frequencies of the TIGIT and PD‐1 double‐negative MAIT (D), MAIT‐like (E) and non‐MAIT (F) cell subsets in the peripheral blood of patients with moderate or severe COVID‐19 and healthy controls. The solid bars represent medians of 14, 18 and 17 determinations, respectively. The boxes indicate the interquartile ranges; the whiskers represent the variability of the minimum, maximum and any outlier data points compared to the interquartile range. The middle square within the box represents the mean value. Significant differences with *p* < 0.01*** are indicated.

The frequency of TIGIT/PD‐1 double‐positive MAIT‐like cells was significantly higher in severe patients compared to the healthy population (Figure 7B). In contrast, the frequency of TIGIT/PD‐1 double‐negative MAIT‐like cells was significantly lower in ICU patients compared to healthy individuals (Figure 7E). Furthermore, the frequency of TIGIT/PD‐1 double‐positive MAIT cells was significantly increased in ICU patients compared to both healthy individuals and IDU patients (Figure 7A).

## 4 Discussion

MAIT cells are known to be key actors in pulmonary mucosal immunity and mucosal tissue repair and are involved in the immune response against numerous respiratory bacterial and viral pathogens [29]. Further research is required to determine the protective and potentially immunopathogenic roles that MAIT cells play during severe viral diseases.

Studies demonstrated that viral infections trigger the activation of unconventional T cells, particularly in the bloodstream and lungs [25]. MAIT cell activation by viruses is TCR independent and cytokine dependent. During both acute and chronic viral infections, the frequency of MAIT cells in the peripheral blood decreases with an apparent enrichment in the airways, whereas the expression of HLA‐DR, PD‐1, CD38 and CD69 is upregulated [9, 12, 29]. The loss of MAIT cells is much more pronounced than that of other T cell subsets in percentage and absolute counts [9, 12, 29]. Interestingly, in severe COVID‐19 patients, higher MAIT cell activation levels were associated with and predictive of mortality [5, 30]. This does not rule out the potential for MAIT cells to provide some level of protection in most non‐severe or mild cases of COVID‐19.

Consistent with previous research, in our study, MAIT cells were significantly depleted in the peripheral blood of both moderate and severe patients compared to healthy controls [9, 12, 29, 31, 32]. This observation suggests that the reduction in MAIT cell frequency is characteristic of COVID‐19 and is due to their recruitment to the airways (endotracheal aspirates, bronchoalveolar fluid and pleural effusions) of the inflamed lungs rather than their physical loss during active disease [9, 12]. In addition, no significant alterations in the frequencies of MAIT‐like and non‐MAIT cells were detected in COVID‐19 patients compared to healthy controls, which aligns with previous studies [12, 31].

Regarding the relative CD8 expression of MAIT cells, we found no differences between the examined groups. The level of CD8 expression was constant in both healthy and ill subjects and consistently lower in each group than in MAIT‐like and non‐MAIT cells. The role of the CD8 co‐receptor on MAIT cells has been elucidated in previous studies, highlighting its significance in antigen responsiveness and subset differentiation. Souter et al. demonstrated that CD8 enhances MR1 binding and cytokine production by MAIT cells, contributing to their functional versatility [33]. Similarly, Dias et al. identified transcriptionally and functionally distinct CD8+ and double‐negative MAIT cell subsets, with CD8+ subsets exhibiting higher cytotoxic potential and reduced propensity for apoptosis [34]. These findings provide a context for our observation that the consistently low CD8 expression levels on MAIT cells in COVID‐19 patients and healthy controls may reflect a preserved regulatory role rather than cytotoxic functionality, consistent with their expected immunological role during systemic infections such as COVID‐19.

The available data suggest that the MAIT cell response to viral infections is predominantly orchestrated by innate cytokines produced by cells responding to the virus, with minimal to no direct involvement of MR1; moreover, the effector functions induced by these cytokines are relatively limited, characterised by the upregulation of CD69, granzyme B and IFN‐γ [35]. Consequently, hypothesising the critical role that MAIT cells play in the immune response to COVID‐19, our research endeavours have been focused on investigating the influential role of immune checkpoint pathways in modulating MAIT cell activity and functionality during viral infection. Based on the available literature to date, our study is the first work to investigate a range of immune checkpoint markers of MAIT cells in moderate and severe acute COVID‐19 patients' peripheral blood.

Yang et al. identified a critical mechanism in severe COVID‐19 where suppressive monocytes reduce the number and impair the function of MAIT cells via increased IL‐10 production [32]. This interaction, exacerbated by microbial co‐infections, aligns with our observations of altered immune checkpoint expressions in MAIT cells, suggesting potential targets to enhance immune responses in severe cases.

In our study with all patients being in a cytokine storm, in severe COVID‐19 patients, circulating MAIT cells showed a significant decrease in the expression of the activating immune checkpoint receptor CD226, whereas the frequency of TIGIT/PD‐1 double‐positive MAIT cells was significantly increased in severe patients compared to both healthy individuals and moderate patients. We could reveal no difference in the examined immune checkpoint molecules expressed by MAIT cells between healthy individuals and moderate patients, suggesting that the immune checkpoint regulation is unfavourably altered, specifically in severe cases. The decreased activation and continuously increasing inhibition as the disease proceeds reflect a growing attempt to mitigate the MAIT response, yet the need for intensive care in severe patients implies insufficiency of this mitigation. Kammann et al. showed that some patients recovering from severe COVID‐19 have an exhausted MAIT cell compartment characterised by high PD‐1 expression and poor responsiveness (IFN‐γ and granzyme B expression) to cytokine stimulation, which can suggest their role in the long‐term effects of the disease [36].

MAIT‐like and non‐MAIT cells demonstrated a significant increase in the expression of the inhibitory immune checkpoint molecule PD‐1 compared to healthy individuals. This upregulation of PD‐1 may serve as a mechanism to mitigate the excessive activity of these cells in both moderate and severely ill individuals.

Nevertheless, since we found a significant increase in non‐MAIT cell frequency in the CD8+ T cell gate in deceased ICU patients compared to patients with severe disease who survived COVID‐19 infection, we can conclude that changes in other immune cell populations not directly associated with mucosal sites may also play a crucial role in how the hospitalisation will end for ICU patients.

SARS‐Cov‐2 infection triggers an inflammatory response that can lead to debilitating illness, cytokine storms and eventually death. Emerging studies into various non‐conventional T cell subsets involved in both the pathogenesis and resolution of COVID‐19 revealed interesting information about how these cells function during a viral infection. However, it remains unclear how the underlying immune regulation influences their function.

Nevertheless, our study also bears several limitations. First, the relatively small sample size limits the statistical power and prevents further desired analyses. Second, our experiments focused on peripheral blood cells, potentially overlooking organ‐specific immune processes in specific microenvironments. Third, the number of PBMCs available for experiments was limited, thus restricting the range of experiments we could perform, including functional assays, which could have added substantial value to the study. Fourth, longitudinal studies were not conducted to assess immune cell responses over time, which could provide valuable insights into immune changes. Fifth, the loss of CD161 expression has been reported during chronic viral infections, potentially due to sustained immune activation and exhaustion, as well as phenotypic changes in T cell populations. Such downregulation may also influence the identification and characterisation of MAIT cells and related subpopulations. This phenomenon could have impacted our observations. Finally, our cohort's limited number of deceased patients restricts a meaningful correlation between disease outcomes and immunological responses.

## 5 Conclusion

Our data suggest that MAIT cell phenotypes are markedly altered in patients with COVID‐19, with signs of an attempt to decrease activation and enhance inhibition during the disease course, as shown by the immune checkpoint expression profiles. Insufficient MAIT cell mitigation might be in the background of more severe cases, besides the potential role of other immune cell populations.

## Conflicts of Interest

The authors declare no conflicts of interest.

## Data Availability

The data that support the findings of this study are available from the corresponding author upon reasonable request.

## References

[1] M. Karmakar , P. M. Lantz , and R. Tipirneni , “Association of Social and Demographic Factors With COVID‐19 Incidence and Death Rates in the US,” JAMA Network Open (2021). 10.1001/jamanetworkopen.2020.36462 PMC7846939 33512520

[2] E. Pairo‐Castineira , S. Clohisey , L. Klaric , et al., “Genetic Mechanisms of Critical Illness in COVID‐19,” Nature 591 (2021): 92–98. 33307546 10.1038/s41586-020-03065-y

[3] B. Hu , S. Huang , and L. Yin , “The Cytokine Storm and COVID‐19,” Journal of Medical Virology 93 (2021): 250–256. 32592501 10.1002/jmv.26232 PMC7361342

[4] C. Herr , S. Mang , B. Mozafari , et al., “Distinct Patterns of Blood Cytokines Beyond a Cytokine Storm Predict Mortality in Covid‐19,” Journal of Inflammation Research 14 (2021): 4651–4667. 34552347 10.2147/JIR.S320685 PMC8451220

[5] J. K. Sandberg , E. Leeansyah , M. A. Eller , B. L. Shacklett , and D. Paquin‐Proulx , “The Emerging Role of MAIT Cell Responses in Viral Infections,” Journal of Immunology 211 (2023): 511–517. 10.4049/jimmunol.2300147 PMC10421619 37549397

[6] E. W. Meermeier , C. L. Zheng , J. G. Tran , et al., “Human Lung‐Resident Mucosal‐Associated Invariant T Cells Are Abundant, Express Antimicrobial Proteins, and Are Cytokine Responsive,” Communications Biology (2022). 10.1038/s42003-022-03823-w PMC9463188 36085311

[7] Y. Long and H. TSC , “MAIT Cells in Respiratory Viral Infections in Mouse and Human,” Critical Reviews in Immunology 41 (2021): 19–35. 35381137 10.1615/CritRevImmunol.2021040877 PMC7612767

[8] J. E. Ussher , C. B. Willberg , and P. Klenerman , “MAIT Cells and Viruses,” Immunology & Cell Biology 96 (2018): 630–641. 29350807 10.1111/imcb.12008 PMC6055725

[9] Y. Jouan , A. Guillon , L. Gonzalez , et al., “Phenotypical and Functional Alteration of Unconventional T Cells in Severe COVID‐19 Patients,” Journal of Experimental Medicine 217 (2020). 10.1084/jem.20200872 PMC7472174 32886755

[10] R. Lamichhane , M. Schneider , S. M. de la Harpe , et al., “TCR‐ or Cytokine‐Activated CD8+ Mucosal‐Associated Invariant T Cells Are Rapid Polyfunctional Effectors That Can Coordinate Immune Responses,” Cell Reports 28 (2019): 3061–3076.e5. 31533031 10.1016/j.celrep.2019.08.054

[11] T. S. C. Hinks , E. Marchi , M. Jabeen , et al., “Activation and in Vivo Evolution of the MAIT Cell Transcriptome in Mice and Humans Reveals Tissue Repair Functionality,” Cell Reports 28 (2019): 3249–3262.e5. 31533045 10.1016/j.celrep.2019.07.039 PMC6859474

[12] T. Parrot , J. B. Gorin , A. Ponzetta , et al., “MAIT Cell Activation and Dynamics Associated With COVID‐19 Disease Severity,” Science Immunology 5 (2020): 1–14. 10.1126/sciimmunol.abe1670 PMC7857393 32989174

[13] J. Shi , J. Zhou , X. Zhang , et al., “Single‐Cell Transcriptomic Profiling of MAIT Cells in Patients With COVID‐19,” Frontiers in Immunology 12 (2021): 1–11. 10.3389/fimmu.2021.700152 PMC8363247 34394094

[14] E. Catafal‐Tardos , M. V. Baglioni , and V. Bekiaris , “Inhibiting the Unconventionals: Importance of Immune Checkpoint Receptors in γδ T, MAIT, and NKT Cells,” Cancers (Basel) 13 (2021). 10.3390/cancers13184647 PMC8467786 34572874

[15] M. Meggyes , D. U. Nagy , B. Szigeti , et al., “Investigation of Mucosal‐Associated Invariant T (MAIT) Cells Expressing Immune Checkpoint Receptors (TIGIT and CD226) in Early‐Onset Preeclampsia,” European Journal of Obstetrics, Gynecology, and Reproductive Biology 252 (2020): 373–381. 32682212 10.1016/j.ejogrb.2020.06.031

[16] M. S. Rha , H. W. Jeong , J. H. Ko , et al., “PD‐1‐Expressing SARS‐CoV‐2‐Specific CD8+ T Cells Are Not Exhausted, but Functional in Patients With COVID‐19,” Immunity 54 (2021): 44–52.e3. 33338412 10.1016/j.immuni.2020.12.002 PMC7834198

[17] E. Lozano , M. Dominguez‐Villar , V. Kuchroo , and D. A. Hafler , “The TIGIT/CD226 Axis Regulates Human T Cell Function,” Journal of Immunology 188 (2012): 3869–3875. 10.4049/jimmunol.1103627 PMC3324669 22427644

[18] Z. Huang , G. Qi , J. S. Miller , and S. G. Zheng , “CD226: An Emerging Role in Immunologic Diseases,” Frontiers in Cell and Development Biology 24 (2020): 564. 10.3389/fcell.2020.00564 PMC7396508 32850777

[19] K. E. Pauken and E. J. Wherry , “TIGIT and CD226: Tipping the Balance Between Costimulatory and Coinhibitory Molecules to Augment the Cancer Immunotherapy Toolkit,” Cancer Cell 26 (2014): 785–787. 25490444 10.1016/j.ccell.2014.11.016

[20] S. Cappanera , M. Palumbo , S. H. Kwan , et al., “When Does the Cytokine Storm Begin in COVID‐19 Patients? A Quick Score to Recognize It,” Journal of Clinical Medicine 10 (2021): 1–12. 10.3390/jcm10020297 PMC7830161 33467466

[21] R Core Team , R: A Language and Environment for Statistical Computing (R Foundation for Statistical Computing, 2022), https://www.R‐project.org/.

[22] J. R. Fergusson , M. H. Hühn , L. Swadling , et al., “CD161intCD8+ T Cells: A Novel Population of Highly Functional, Memory CD8+ T Cells Enriched Within the Gut,” Mucosal Immunology 9 (2016): 401–413. 26220166 10.1038/mi.2015.69 PMC4732939

[23] E. Merlini , M. Cerrone , B. van Wilgenburg , et al., “Association Between Impaired vα7.2+cd161++cd8+ (MAIT) and vα7.2+cd161‐cd8+ t‐Cell Populations and Gut Dysbiosis in Chronically HIV‐and/or HCV‐Infected Patients,” Frontiers in Microbiology 10 (2019). 10.3389/fmicb.2019.01972 PMC6722213 31555223

[24] D. Park , H. G. Kim , M. Kim , et al., “Differences in the Molecular Signatures of Mucosal‐Associated Invariant T Cells and Conventional T Cells,” Scientific Reports 9 (2019). 10.1038/s41598-019-43578-9 PMC6506535 31068647

[25] P. E. Lo , A. De Gaetano , G. Pioggia , and S. Gangemi , “Comprehensive Analysis of the ILCs and Unconventional T Cells in Virus Infection: Profiling and Dynamics Associated With COVID‐19 Disease for a Future Monitoring System and Therapeutic Opportunities,” Cells 11 (2022): 11. 10.3390/cells11030542 PMC8834012 35159352

[26] T. Abdelaal , V. van Unen , T. Höllt , F. Koning , M. J. T. Reinders , and A. Mahfouz , “Predicting Cell Populations in Single Cell Mass Cytometry Data,” Cytometry. Part A 95 (2019): 769–781. 10.1002/cyto.a.23738 PMC6767556 30861637

[27] V. Thomas‐Vaslin , H. K. Altes , R. J. de Boer , and D. Klatzmann , “Comprehensive Assessment and Mathematical Modeling of T Cell Population Dynamics and Homeostasis,” Journal of Immunology 180 (2008): 2240–2250. 10.4049/jimmunol.180.4.2240 18250431

[28] C. Taswell , “Limiting Dilution Assays for the Determination of Immunocompetent Cell Frequencies. I. Data Analysis,” Journal of Immunology 126 (1981): 1614–1619, 10.4049/jimmunol.126.4.1614. 7009746

[29] H. Flament , M. Rouland , L. Beaudoin , et al., “Outcome of SARS‐CoV‐2 Infection Is Linked to MAIT Cell Activation and Cytotoxicity,” Nature Immunology 22 (2021): 322–335. 33531712 10.1038/s41590-021-00870-z

[30] J. Youngs , N. M. Provine , N. Lim , et al., “Identification of Immune Correlates of Fatal Outcomes in Critically Ill COVID‐19 Patients,” PLoS Pathogens 17 (2021): 1–28, 10.1371/journal.ppat.1009804. PMC8445447 34529726

[31] S. Deschler , J. Kager , J. Erber , et al., “Mucosal‐Associated Invariant T (MAIT) Cells Are Highly Activated and Functionally Impaired in COVID‐19 Patients,” Viruses 13 (2021): 1–18. 10.3390/v13020241 PMC7913667 33546489

[32] Q. Yang , Y. Wen , F. Qi , et al., “Suppressive Monocytes Impair MAIT Cells Response via IL‐10 in Patients With Severe COVID‐19,” Journal of Immunology 207 (2021): 1848–1856. 10.4049/jimmunol.2100228 34452933

[33] M. N. T. Souter , W. Awad , S. Li , et al., “CD8 Coreceptor Engagement of MR1 Enhances Antigen Responsiveness by Human MAIT and Other MR1‐Reactive T Cells,” Journal of Experimental Medicine 219 (2022). 10.1084/jem.20210828 PMC9424912 36018322

[34] J. Dias , C. Boulouis , J. B. Gorin , et al., “The CD4‐CD8‐MAIT Cell Subpopulation Is a Functionally Distinct Subset Developmentally Related to the Main CD8+ MAIT Cell Pool,” Proceedings of the National Academy of Sciences of the United States of America 115 (2018): E11513–E11522. 30442667 10.1073/pnas.1812273115 PMC6298106

[35] T. S. C. Hinks and X. W. Zhang , “MAIT Cell Activation and Functions,” Frontiers in Immunology 11 (2020). 10.3389/fimmu.2020.01014 PMC7267072 32536923

[36] T. Kammann , J.‐B. Gorin , T. Parrot , et al., “Dynamic MAIT Cell Recovery After Severe COVID‐19 Is Transient With Signs of Heterogeneous Functional Anomalies,” Journal of Immunology 20 (2023): 389–396. 10.4049/jimmunol.2300639 PMC10784727 38117799

